# Plant Detection in RGB Images from Unmanned Aerial Vehicles Using Segmentation by Deep Learning and an Impact of Model Accuracy on Downstream Analysis

**DOI:** 10.3390/jimaging11010028

**Published:** 2025-01-20

**Authors:** Mikhail V. Kozhekin, Mikhail A. Genaev, Evgenii G. Komyshev, Zakhar A. Zavyalov, Dmitry A. Afonnikov

**Affiliations:** 1 Institute of Cytology and Genetics, Siberian Branch of Russian Academy of Sciences, 630090 Novosibirsk, Russia; 2 Kurchatov Center for Genome Research, Institute of Cytology and Genetics, Siberian Branch of Russian Academy of Sciences, 630090 Novosibirsk, Russia; 3 Department of Mechanics and Mathematics, Novosibirsk State University, 630090 Novosibirsk, Russia; 4 GeosAero LLC, 440000 Penza, Russia

**Keywords:** crop, field image, plant counting, UAV, deep learning, semantic segmentation, image texture analysis

## Abstract

Crop field monitoring using unmanned aerial vehicles (UAVs) is one of the most important technologies for plant growth control in modern precision agriculture. One of the important and widely used tasks in field monitoring is plant stand counting. The accurate identification of plants in field images provides estimates of plant number per unit area, detects missing seedlings, and predicts crop yield. Current methods are based on the detection of plants in images obtained from UAVs by means of computer vision algorithms and deep learning neural networks. These approaches depend on image spatial resolution and the quality of plant markup. The performance of automatic plant detection may affect the efficiency of downstream analysis of a field cropping pattern. In the present work, a method is presented for detecting the plants of five species in images acquired via a UAV on the basis of image segmentation by deep learning algorithms (convolutional neural networks). Twelve orthomosaics were collected and marked at several sites in Russia to train and test the neural network algorithms. Additionally, 17 existing datasets of various spatial resolutions and markup quality levels from the Roboflow service were used to extend training image sets. Finally, we compared several texture features between manually evaluated and neural-network-estimated plant masks. It was demonstrated that adding images to the training sample (even those of lower resolution and markup quality) improves plant stand counting significantly. The work indicates how the accuracy of plant detection in field images may affect their cropping pattern evaluation by means of texture characteristics. For some of the characteristics (GLCM mean, GLRM long run, GLRM run ratio) the estimates between images marked manually and automatically are close. For others, the differences are large and may lead to erroneous conclusions about the properties of field cropping patterns. Nonetheless, overall, plant detection algorithms with a higher accuracy show better agreement with the estimates of texture parameters obtained from manually marked images.

## 1. Introduction

Unmanned aerial vehicle (UAV)-based crop monitoring is one of the most important technologies for controlling plant growth in modern precision agriculture [1,2]. The high mobility of UAVs allows for the monitoring of large fields and the collection of data from all areas. Due to the ability to use different sensors, this technology has found a wide range of applications in agriculture [3,4] and field plant phenomics [5,6]. These applications include estimation of the soil moisture content [7], weed detection [8], and assessment of the leaf area index (LAI) and plant biomass [9,10]. Methods have been developed to monitor plant nitrogen status [11] and plant height [12], to detect pathogens [13], and to evaluate plant phenology in the field [14,15]. A promising approach is to employ data obtained from UAV monitoring for the yield prediction of crops [16,17,18].

One of the important and common tasks in field monitoring is plant stand counting in images obtained from UAVs [19]. The number of plants per unit area is closely related to yield. The evaluation of these parameters can be performed at different stages of plant development. At the early stages, it characterizes germination and allows for the planning of subsequent agronomic measures to achieve the highest yield. The estimation of plant numbers via ground observations is labor-intensive and time-consuming. Visual inspection is prone to human error and subjectivity. Even the use of hand-held digital cameras allows only small areas of fields to be inspected [20]. Methods based on RGB image acquisition by means of a UAV are much more productive. As a rule, they include several basic steps [6,21,22,23]: (1) the UAV flight and raw-image capture, (2) the stitching of raw images into a single orthomosaic, (3) the development of a digital-image-processing method to identify plants in the orthomosaic, and (4) subsequent analysis for the formulation of recommendations to agronomists. The main efforts of researchers in the field of image analysis are focused on step 3: techniques for fast and accurate plant identification [19,24].

Two types of methods are used for plant identification based on the analysis of digital orthophoto images: those involving computer vision and those based on deep machine learning [19]. The first type necessitates binarization/segmentation algorithms, which are typically implemented via a combination of R, G, and B components to compute indices in order to distinguish plant pixels from soil pixels [21,25,26,27]. During the postprocessing of plant areas in the images, the separation of objects and the removal of noise (usually small objects) take place. Several approaches are employed for these purposes: the evaluation of an object’s shape/size features and machine learning algorithms [21], peak detection in plant rows [26,27,28], and searching for statistical correlations [29]. The image objects filtered by postprocessing are thought to correspond to plants, and their counting is then performed.

Deep learning methods are based on networks characterized by a multilayered architecture where subsequent layers utilize the output of a preceding layer as an input to extract features related to the analyzed objects [24,30,31]. These approaches enable the automatic extraction of image features with regression or classification within a single pipeline, trained simultaneously from end to end [32].

Deep machine learning techniques can solve three of the most frequent image processing problems: image classification, image segmentation, and searching for objects in an image [33,34,35]. The two latter approaches are used to count plants in UAV images [24,36]. When the segmentation problem is being solved by deep machine learning methods, no data preprocessing or construction of various indices is required because it is used in computer vision methods. Necessary plant parameters are extracted automatically.

For semantic segmentation, architectures based on convolutional neural networks (CNNs) are currently used [37]: fully convolutional networks, convolutional networks with graphical models, encoder–decoder-based models, and some others. These methods have been successfully utilized in plant counting problems using different network topologies. Fully convolutional networks have been employed for plant identification in field images of corn and strawberry [38] and for plant and weed identification [39]. U-Net-based topologies have been used to count maize plants at an early developmental stage [40], to identify plants in high-elevation ecosystems [41], and to recognize a maize plant in the field [42]. A network with a SegNet architecture has been utilized to count bolls of cotton to estimate its yield [43]. Note that semantic segmentation approaches require the further processing of the neural network results in order to identify individual plants in the images [38]. They are most suitable for counting plants at the early stages of development, while the contours of individual plants in the images are not touching each other.

Instance segmentation methods are convenient for solving the problem of counting plants in an image because they enable an investigator to select image regions corresponding to different plants. These techniques are actively used in tasks of object extraction from an image [37]. One of the most popular network architectures for instance segmentation is Region CNN (R-CNN) [44] and its modifications. A Mask R-CNN network has been used for counting the medicinal plants *Lamiophlomis rotata* in mountain landscapes [45] as well as potato and lettuce plants [46]. Faster R-CNN networks have been employed for the counting of maize, sugar beet, sunflower plants [47], corn plants [48], and potato plants [49] in field images.

Object detection methods are also actively used for plant counting. In particular, these are networks based on You Only Look Once (YOLO) architectures [50]. They have a higher speed and have been applied to count cotton [51], sorghum [52], and maize [53] plants. Several neural network architectures have been used for the detection of citrus trees in images [54]. Detection and instance segmentation approaches tend to identify individual plants more accurately, even if the plants touch each other. Nonetheless, they are more computationally and memory-demanding [35].

It should be noted that the identification of plants in images is not the only purpose of such projects for agronomists but serves as a basis for the subsequent assessment of crop characteristics in order to select optimal agronomic treatments. The mean absolute error and coefficients of correlation between a predicted number of plants and the true number of plants give an estimate of the bias in plant density when machine learning algorithms are applied [21]. Other characteristics include sowing uniformity [55] and row regularity for weed identification [56,57,58].

One approach to the estimation of the uniformity of objects in an image is to use texture features [59,60,61]. In crop analysis, texture parameters are employed in particular for between- and within-crop-row weed detection [56,57]. Nevertheless, how the accuracy of plant identification by machine learning affects the characteristics of plant arrangement remains unclear.

Here, a method is presented for detecting the plants of five species in images acquired from a UAV on the basis of image segmentation via deep learning algorithms (CNNs). Twelve orthomosaics were collected and marked at several sites in Russia to train and test neural network algorithms. Additionally, 17 external datasets from the Roboflow service were utilized to extend image sets. Finally, we compared several texture features for manually assessed and neural-network-estimated plant masks.

## 2. Materials and Methods

### 2.1. Image Acquisition and Construction of Orthomosaics

**Figure 1:**
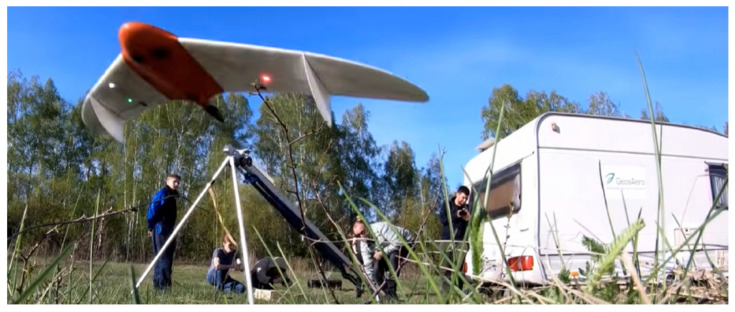
A UAV launching from the launcher.

The locations of the fields (Penza Oblast, Krasnodar Krai, and Stavropol Krai, Russia) are given in Table S1 (Supplementary Materials). Image acquisition in each field was performed within 1 day by means of three UAVs of the Geoscan 201 Agrogeodesia model of the flying wing type (Figure 1). The UAVs are equipped with a Sony RX1R II RGB camera (Sony Corporation, Tokyo, Japan) with a resolution of 42.4 Mpix. The flight altitude was 50 m. Each UAV is capable of imaging 8000 ha per day. The flights and aerial image acquisition were carried out by GeosAero LLC (Penza, Russia).

Orthomosaics were built from images using Agisoft Metashape Professional (https://www.agisoft.com (accessed on 1 October 2023)). The coordinate system was WGS:84 (EPSG:4326), and the file format was geotiff. Orthomosaics were obtained with a spatial resolution of at least 2 cm/pix.

### 2.2. Image Datasets

#### 2.2.1. Data from Russian Regions During 2019–2023

The main dataset included orthomosaic images of different crops at the early seedling stage from several Russian regions.

**Figure 2:**
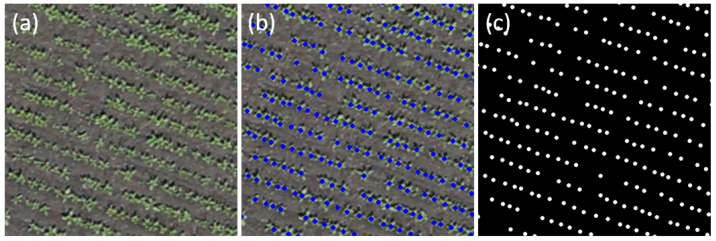
Examples of images for the analysis. (**a**) A fragment of an orthomosaic before markup; (**b**) the same fragment with vector markup applied in QGIS; (**c**) a generated raster mask showing the location of plant centers.

The process of markup of these images was carried out in the QGIS Desktop software (https://qgis.org (accessed on 1 October 2023)). Plant centers were manually marked, and the resulting images were saved in vector format as a markup file (Figure 2). Because the scale of the orthomosaics differed in some cases by almost a factor of 2, all orthomosaics were resized to the same scale (1 pix = 1 cm) before analysis. This procedure was performed using the resize() function of the Python Imaging Library by means of an appropriate normalization factor. Because the input of the neural network has to be supplied with a markup image in raster format, raster masks were generated from the vector data at the same scale. The mask included the background (black color) and plant centers (white circles with a radius of 4 cm).

The list of 12 orthomosaic images is given in Table 1. The total area of the crop fields in the orthomosaics was 23.494 ha, and the total number of plants was 610,067.

Thus, the acquired images provide a large amount of data for the training of deep learning neural network algorithms.

#### 2.2.2. Public Datasets

Additional image datasets were included in this work to extend training sets. They were obtained from the Roboflow service (https://universe.roboflow.com (accessed on 25 November 2023)). The datasets were searched for keywords (crop name, growth stages, field survey, and UAV) in November 2023. In the retrieved datasets, duplicates and images of markup with insufficient quality were excluded. As a result, 14 datasets for five plant species were selected: sugar beet, corn, sunflower, potato, and tobacco. The list of datasets is summarized in Table S2 (Supplementary Material).

Some images were of a high spatial resolution, ~0.42 cm/pix [28] (marked with an asterisk in Table S2). These images were used to build orthomosaics (UBONN_Sb1_2015, UBONN_Sb2_2015, and UBONN_Sb3_2015), which were resized to a common scale and marked as described above.

The rest of the images were converted to a scale of 1 pix = 1 cm. Note that information on spatial resolution was not available for most of the additional images with a lower resolution. Therefore, information about row spacing for different crops was employed to determine an image scale (Table S3, Supplementary Material). The added images contain plants marked by rectangles. Plant centers were identified as the centers of rectangles and marked with a circle having a radius of 4 cm in these images. Weed plants were excluded from these datasets. Based on this marking, a raster mask for each image was generated.

Thus, in this study, the total image sample consisted of 7456 field images in which 362,230 plants of five agricultural crops were marked.

#### 2.2.3. Data Stratification

The data were collected in Russia (Table 1) and the additional data (Table S2, Supplementary Material) were stratified into four datasets. Three datasets (HQ1, HQ2, and HQ3) consisted of orthomosaics; most of them were obtained in Russia in 2019–2023 via a unified acquisition protocol. They have an initial spatial resolution of at least 2 cm/pix, and their labeling was performed manually. They represent high-quality markup images. The fourth dataset (LQ) includes individual frames acquired with the help of UAVs or ground-based imaging. It contains public images from the Roboflow service without the additional manual correction of plant centers in the markup files. These four datasets were balanced in terms of the number of images. The list of included datasets is given in Table 2.

### 2.3. Neural Network Architecture and Learning Algorithms

**Figure 3:**
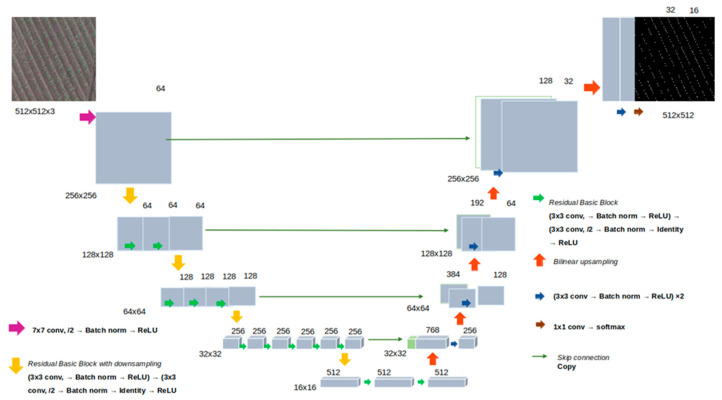
The architecture of the U-Net network used in this work for plant identification.

In the analysis, each image was divided into 512 × 512 px tiles using the RandomCrop(512,512) function of the PyTorch 2.0.1 package. Tile images served as the input for a neural network. The U-Net architecture with the ResNet-18 encoder [62] was chosen as a baseline model. The network structure is shown in Figure 3 and includes an encoder part and a decoder part. The encoder consists of convolution layers, with each layer performing convolution operations, normalization batch, ReLU activation functions, and subsampling operations. The output of the encoder has a dimensionality of 512 × 16 × 16. The U-Net decoder is composed of upsampling (backpropagation), convolution layers, concatenation with the corresponding encoder layers, and normalization. The outputs of each decoder layer are concatenated with corresponding encoder layers of the same dimensionality; the last decoder layer has a dimensionality of 512 × 512, corresponding to a single-channel segmentation mask. Additionally, encoders with ResNet-34 and ResNet-50 architectures were used. They have different numbers of layers and a larger number of parameters. The characteristics of the encoders are given in Table S4 (Supplementary Material).

The Adam adaptive optimizer [63] was utilized to fit network parameters. The learning rate decreased linearly from 10^−4^ to 10^−6^ with a batch size of 8. To optimize model weights, the combined loss function DiceCE was used. It is defined as the sum of Cross Entropy [62] and Dice [64]. Cross Entropy evaluates the quality of a classification. Dice computes a measure of similarity between a predicted mask and the true segmentation mask.

In the training process, we carried out the procedure of image augmentation using the Albumentations library [65]: rotate an image by a random angle in the range from 0 to 90° (method Rotate()), randomly change the scale by a value below 30% (method RandomScale()), randomly change brightness and contrast (methods RandomBrightnessContrast() and RandomGamma()), and perform random vertical and horizontal mapping (methods HorizontalFlip() and VerticalFlip()). Data processing was implemented in Python 3.12 and the Pytorch 2.0.1 framework.

The network model was trained for 100 epochs. The best model was selected based on the metrics computed for a validation sample.

Finally, after image segmentation, plant contours were determined in the predicted mask images by the cv2.findContours() method from the OpenCV library [66]. Segmented objects whose area was smaller than a given threshold were excluded as possible noise.

Several experiments were conducted to evaluate the influence of network structure and a combination of datasets in the training/validation and test samples on the accuracy of plant recognition (Table 3). In experiments code-named “HR,” validation and testing were based on high-resolution images. In the RN18-LR experiment, low-resolution external images served as the training sample. Experiments RN34-HR-LR and RN50-HR-LR3 differed (from the two other experiments) in that encoders with a larger number of parameters were used: ResNet-34 and ResNet-50 (Table 3).

### 2.4. Evaluating Accuracy of Plant Identification

The *IoU* (intersection over union) metric was employed to assess segmentation quality for true *X* and predicted *Y* contours:

$$IoU=\frac{X\cap Y}{X\cup Y}.$$

To evaluate the quality of model performance, we used Pearson’s correlation coefficient *r*, Spearman’s correlation coefficient *r_s_*, mean absolute error *MAE*, and mean absolute percentage error *MAPE*, which were calculated via the formulas

$$MAE=\frac{1}{n}\sum_{i=1}^{n}\left|x_{i}-y_{i}\right|,$$

$$MAPE=\frac{100}{n}\sum_{i=1}^{n}\frac{\left|x_{i}-y_{i}\right|}{y_{i}}$$

where *x_i_* and *y_i_* are the number of plants in the *i*th orthomosaic fragments obtained by manual markup and a neural network, respectively; *n* is the number of such tiles. We utilized non-overlapping tiles of 20 × 20 m in size.

### 2.5. The Downstream Analysis of the Processed Orthomosaics

The evaluation of several texture characteristics was carried out as the downstream analysis of the crop image masks obtained either manually or by neural networks. These characteristics are dependent on a mutual arrangement of plants in the images, its regularity, proximity, and other factors. Second-order texture characteristics that are determined on the basis of the Gray Level Co-occurrence Matrix (GLCM) and Gray Level Run-length Matrix (GLRM) [67,68,69] were chosen for the analysis.

Initially, we evaluated 10 characteristics; however, most of them turned out to be highly correlated. Therefore, four texture features were selected for our analysis: GLCM mean, GLCM correlation, GLRM long run, and GLRM run ratio. They are defined in Table S5 (Supplementary Material). Eight main directions were used to calculate texture features for neighboring pixels (GLCM features) and for a series (GLRM features): up, down, left, right, and four diagonal directions [70].

Texture characteristics were estimated for images of the test sample obtained in Russia (dataset HR3 except for UBONN_Sb3_2015, Table 2). Nonoverlapping tiles of size 1000 × 1000 px from orthomosaics were employed to evaluate texture characteristics. Statistical associations were evaluated between true mask tiles (manual markup) and tiles marked by a neural network. Statistical analysis was performed with the help of the numpy library of the Python language.

## 3. Results

### 3.1. The Evaluation of the Accuracy of Plant Identification in Different Experiments

**Figure 4:**
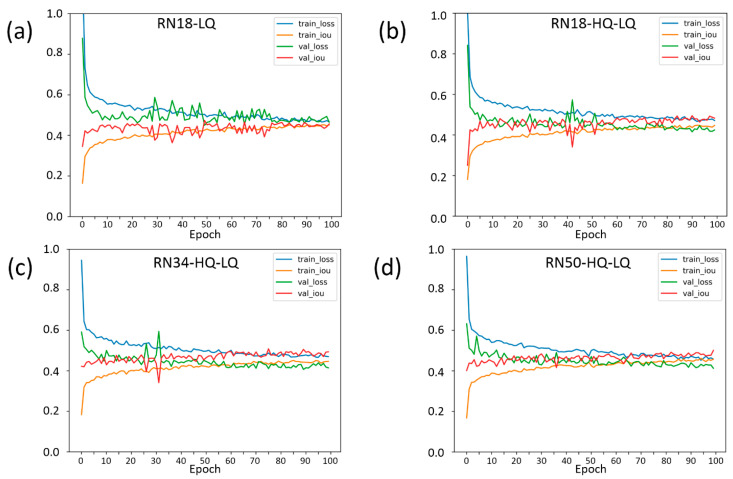
The learning curves of the models corresponding to the experiments: (**a**) RN18-LQ; (**b**) RN18-HQ-LQ; (**c**) RN34-HQ-LQ; and (**d**) RN50-HQ-LQ. On the X-axis, the ID numbers of epochs during training are plotted. The Y-axis shows parameters characterizing the magnitude of error obtained with the training and validation samples (see the panels in the top-right corner of the graphs). Blue curve: change in the loss function on the training sample; green curve: change in the loss function on the validation sample; yellow curve: change in the *IoU* metric on the training sample; red curve: change in the *IoU* metric on the validation sample.

Changes in the loss function and *IoU* for plant identification in training and validation samples during training are shown in Figure 4 for four experiments.

As presented in the graph in Figure 4a, only on a low-resolution image sample (RN18-LQ) network topology does the training ResNet-18 network yield high-amplitude fluctuations in the loss function and *IoU* at the initial stage of the training process. The variation in these parameters stabilized around epoch 75 (Figure 4a). In an experiment with the same network architecture and both high- and low-resolution data as the training sample (RN18-HQ-LQ, Figure 4b), the loss and *IoU* stabilized at 50 epochs. For the RN34-HQ-LQ experiment (Figure 4c), where a network architecture with a larger number of parameters was used, ResNet-34, the loss, and *IoU* stabilized at epoch 35. The use of the ResNet-50 network architecture and low- and high-resolution images in the training process showed a smooth change in accuracy characteristics without substantial spikes (Figure 4d).

Table 4 summarizes the accuracy assessment metrics for the experiments described in Table 4. The table contains the average values of the metrics for the five orthomosaics included in the test sample. As one can see in the table, the use of low-resolution data and the ResNet-50 encoder yielded the best results (experiment RN50-HQ-LQ): the Pearson’s coefficient of correlation between the number of plants determined manually and the predicted number of plants was greater than 0.98. The parameters *MAPE* and *MAE* for the best model were the smallest. The model is able to segment sunflower, potato, and sugar beet plants at an early stage of growth, regardless of light and other conditions. Nonetheless, Spearman’s correlation coefficient and *IoU* were not the greatest for the RN50-HQ-LQ experiment. In terms of *r*_s_, it was ranked #2 and in terms of *IoU* #3 by value.

A comparison of the accuracy parameters between RN18-HQ and RN18-LQ revealed a difference between the cases of HQ and LQ data used for training. RN18-HR yielded better estimates for *MAPE*, *MAE*, and *IoU*. In the RN18-LQ experiment, these parameters showed lower performance (note that the test dataset contained HQ data only). On the other hand, both correlation coefficients were smaller in the RN18-HR experiment and larger in RN18-LQ. This finding implies that using the LQ dataset decreases the accuracy of finding a plant center (a lower value of *IoU* in all experiments). Nevertheless, the larger number of images in this dataset (Table 3) decreased the variance for the estimate of the number of plants in the image (higher correlation coefficients). Of note, using both the HQ and LQ datasets in training (experiment RN18-HQ-LQ) yielded better plant count estimates (*MAE*, *MAPE*, *r*, and *r*_s_) but a lower *IoU* value in comparison with the RN18-HQ experiment.

**Figure 5:**
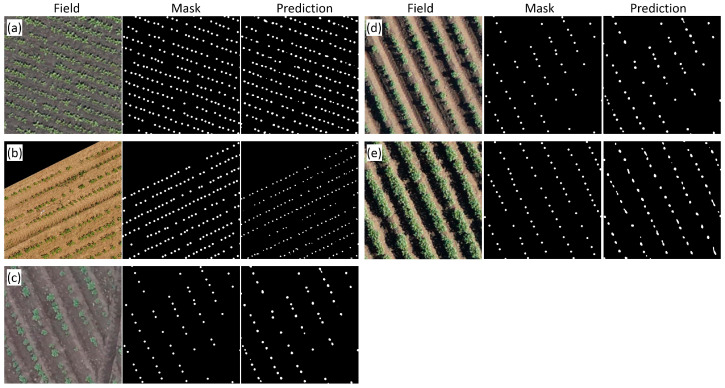
Examples of RN50-HQ-LQ model performance on the test sample for different crops and high-resolution orthomosaics. (**a**) Sugar beet, Beet_marat_1; (**b**) sugar beet, UBONN_Sb3_2015; (**c**) potato, Stavropol_2_7; (**d**) potato, Stavropol_4_0; (**e**) potato, Stavropol_4_9. Images in rows from left to right: original (Field); manual plant marking (Mask); automatic marking by the RN50-HQ-LQ network.

Examples of plant images for different crops and manual and automatic markups are shown in Figure 5. The agreement between automatic and manual markups is high. Nonetheless, some errors could be detected in the masks obtained by means of neural networks: some contours for plants were found to be elongated due to the merging of neighboring plant contours; in some cases, small contours appeared between plants.

Table 5 shows the results of accuracy estimation for the best model (RN50-HQ-LQ) for orthomosaics from the test sample. For most images, accuracy is approximately the same (*MAPE* 1–6%), except for plant recognition for the Stavropol_4_9 orthomosaic. The latter showed lower performance (*MAPE* above 12%). This could be explained by a larger proportion of touching plants in the images obtained from this field (Figure 5e).

We evaluated the performance of the algorithms on a desktop PC with the following configuration: CPU, Intel^®^ Core^TM^ i5-8265U @ 1.60 GHz (Intel Corporation, Santa Clara, CA, USA); GPU, NVIDIA GeForce RTX 2080 12 G (Nvidia Corporation, Santa Clara, CA, USA); GPU environment, CUDA 11.2; OS, Windows 10; software, Python 3.12; and framework, Pytorch 2.0.1. The performance was tested for files larger than 3 GB with an image size of 50,000 × 50,000 px. The estimated computational performance of the RN50-HQ-LQ network is enough to process a large amount of data within a reasonable time, at least 50 ha per hour on the desktop PC with the GPU.

### 3.2. The Influence of Plant Identification Accuracy on Subsequent Analysis

A downstream analysis of the plants in orthomosaics via the evaluation of the texture features was performed next. The plant masks obtained manually and predicted by the networks of the best (RN50-HQ-LQ) and lower (RN18-HQ) performance were used for texture analysis.

A comparison of the mean values for the estimates of the four texture features among different orthomosaics and markups is presented in Table S6 (Supplementary Material). First of all, this table indicates that there are noticeable differences (all of them statistically significant) in mean texture parameters (for the manually marked images) between Beet_marat_1 and the other three datasets. For example, the mean value of the GLCM mean parameter for the Beet_marat_1 dataset is 14.36, while for the other datasets it ranges from 4.5 to 6.5. Similar differences were noted in the other parameters. This is because the Beet_marat_1 dataset has a row spacing of 45 cm, whereas for the other orthomosaics, it is 90 cm (see Table 1). Thus, the estimated texture characteristics may characterize the cropping patterns in the images.

A comparison of the mean values of the features obtained from manually and automatically labeled masks for different datasets revealed that, for the predicted masks, the differences are significant in most cases. Nonetheless, deviations of the mean values of texture features for the more accurate mapping, RN50-HQ-LQ, were overall smaller as compared to the less accurate one, RN18-HQ. For example, for the GLCM mean parameter for the Beet_marat_1 data (14.36), the differences in the estimates between the manually made mask and RN50-HQ-LQ (14.15) are insignificant, whereas in comparison with RN18-HQ (15.00), the differences are significant. Note that for the predicted masks and features based on the length of the gray level series (GLRM longRun and GLRM runRatio), the estimates of the mean based on the manual masks and the predicted ones differ by a factor of almost 2, while the differences in the predicted masks among different datasets are small. This is probably because, unlike the manual marking, in which plant centers are marked with circles that do not touch each other, the marking obtained with the help of neural networks gives elongated areas for plants that often touch each other (Figure 5e). Errors of this kind are more common for the less accurate RN18-HQ network.

**Figure 6:**
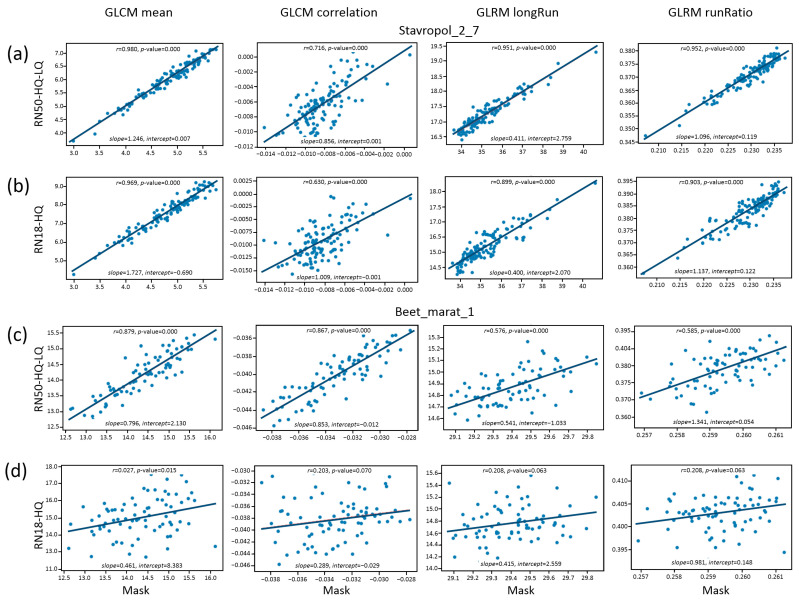
A comparison of crop texture characteristics estimates between markup obtained by the manual approach (X axis) and markup obtained by neural network algorithm (Y axis). The names of characteristics are shown at the top of the figure. (**a**) Stavropol_2_7, prediction by the RN50-HQ-LQ method; (**b**) Stavropol_2_7, prediction by the RN18-HQ method; (**c**) Beet_marat_1, prediction by the RN50-HQ-LQ method; (**d**) Beet_marat_1, prediction by the RN18-HQ method.

The characteristics of the linear relationship between the manual markup (mask) and the predicted markups are shown in Figure 6. The figure suggests that the estimates obtained with neural networks—compared to the manual approach—have systematic biases (the slope and intercept differ from 1.0 and 0, respectively). The correlation coefficients for parameters and datasets in some cases are close to 1.0. In other cases, they deviate strongly from 1.0, even to the point of not being significant (RN18-HQ predictions, the Beet_marat_1 dataset; Figure 6d).

Examples of the masks of some tiles having strong deviations of the GLCM mean parameter for the predicted masks are shown in Figure S1 (Supplementary Material). This figure presents characteristic errors in the marking of orthomosaics by the neural network: the shape of markers differs from the circular one, touching contours are observed, and some markers deviate from a row’s direction. These errors are more pronounced for the markup obtained by the RN18-HQ neural network. Apparently, such errors lead to the deviation of the texture parameters described above.

Visual analysis of the examples of mask images and the results of neural network prediction (Figure S1) demonstrates that for the network with a lower quality of prediction (RN-18-HQ), the images contain more noise (in comparison with a more accurate algorithm, RN50-HQ-LQ). In particular, this noise results from the inclusion of small objects between regularly spaced plant centers. Because of the noise, the RN-18-HQ images appear less homogeneous and regular. This appears to lead to significantly greater distortions in the estimation of the texture characteristics by the RN-18-HQ model (Figure 6). Note also that for the Beet_marat_1 dataset, the plant spacing is smaller than for Stavropol_2_7. This may further amplify the effect of noise when evaluating the texture characteristics (images become less homogeneous). For example, for Stavropol_2_7 images, the less accurate RN-18-HQ network gives estimates of textural characteristics with rather high values of correlation coefficients when compared to the mask, and for the RN-18-HQ and Beet_marat_1 dataset, the correlation coefficients lose significance. In general, it can be assumed that the result of the comparison of texture features is influenced on the one hand by the accuracy of prediction (the presence of noise) and, on the other hand, by the specificity of the plant arrangement pattern (in particular, the distance between rows).

Thus, the effect of estimation accuracy on the subsequent analysis of recognized plant masks strongly depends on which texture parameters are estimated. Nevertheless, overall, for a prediction by a more accurate network, the deviation of mean values of texture features appears to be closer to the deviation obtained via manual partitioning.

## 4. Discussion

In this work, a method was developed for counting plants in field images obtained from a UAV. The neural network approach was used to solve the problem of image segmentation based on the U-Net architecture and the ResNet encoder. The algorithm was tested on several agricultural crops and manifested high accuracy. The estimates of plant-counting accuracy for the best model proved to be comparable to the accuracy of both computer vision-based and deep-learning-based techniques. Computer vision-based approaches achieve relative root mean square error (RRMSE) values between 2 and 4%, depending on the field and crop (maize and sunflower) [25]. When maize plants were counted at different growth stages, RRMSE accuracy estimates of 2–6% were achieved in that study, and more accurately for an earlier stage of plant development. For later stages, when plants begin to touch each other, the accuracy of this method drops severalfold [71]. When plants are counted in images by means of the U-Net neural network, *MAPE* estimates range from 4% to 16%, depending on the field and crop [40]. Using the RiceNet neural network to count rice shoots results in *MAE* values ranging from 3 to 4 [72].

It has been shown that image spatial resolution affects the performance of neural network deep learning algorithms for various tasks [73,74,75,76]. Spatial resolution is critical for an analysis of field images obtained from UAVs for plant counting [23,25,77] and sizing [46]. In the present work, images of different resolutions as well as plant markup quality were employed for the training and testing of neural network algorithms. The HQ set was acquired by a UAV camera via a uniform protocol; the images were assembled into orthophotos followed by manual markup. The LQ set represented fragmented images of fields at different resolutions, whose markup was determined by the recalculation of the positions of the centers of the frames marking the plants. Using only the HQ set for training allows for the more accurate localization of plants in an image. By contrast, using the LQ set, due to the larger number of images, enables us to obtain higher correlations between true and predicted plant counts. Therefore, combining these data for training—despite the differences in resolution and markup quality—significantly improves the accuracy of plant recognition.

Errors in plant identification in the field images inevitably cause biases in crop density and plant spacing estimation [21,55]. The present work illustrates how the precision of plant detection in field images may affect their regularity estimates by means of several texture characteristics. For some characteristics and datasets, the correlation of estimates between images marked manually and images marked automatically is high (GLCM mean, GLRM long run, GLRM run ratio, Stavropol_2_7 dataset). For others, the differences are large and may lead to erroneous conclusions about the properties of field cropping patterns. Thus, overall, methods with a higher accuracy of automatic plant prediction and counting should give estimates of texture parameters close to those derived from manually marked images.

## 5. Conclusions

Segmentation method using deep learning algorithms was developed for detecting and counting plants of five species in RGB images acquired from a UAV. Several CNN models based on the U-Net architecture with different encoders (ResNet-18, ResNet-34, ResNet-50) were implemented. They were trained using orthomosaics with high quality markup obtained at several locations in Russia and additional datasets of various spatial resolutions and markup quality from the Roboflow service. The performance of several neural networks and training datasets was evaluated. The advantage of usage both the high- and low-quality marked images in neural networks training was demonstrated. This strategy yielded better plant count estimates but a lower performance of the plant location in the images (*IoU*). Several texture features characterizing cropping patterns were estimated and compared for manually evaluated and neural-network-estimated plant masks. For some of the texture characteristics (GLCM mean, GLRM long run, GLRM run ratio) the estimates between images marked manually and automatically are close. For others, the differences are large and may lead to erroneous conclusions about the properties of field cropping patterns. In general, plant detection algorithms with a higher accuracy show better agreement with the estimates of texture parameters obtained from manually marked images.

## Figures and Tables

**Table 1:** Orthomosaic images of fields in Russian regions in 2019–2023.

Dataset Name	Crop	Location	Imaging Date	Inter-Row Distance, cm	Resolution GSD cm/px	Area, ha	Number of Plants
Beet_marat_0	Sugar beet	MARAT	2023.06.10	45	1.966	0.924	64,750
Beet_marat_1	Sugar beet	MARAT	2023.06.10	45	1.966	0.924	74,394
Krasnodar	Sugar beet	IVANOVSK	2022.06.23	72	1.778	6.801	75,653
Krasnodar_1	Sunflower	NOVOMINSK	2022.06.23	93	1.032	4.156	92,709
Stavropol_1_7	Sunflower	VINODEL	2019.05.25	70	1.791	1.337	59,044
Stavropol_2_7	Potato	VINODEL	2019.05.25	90	1.874	1.336	30,518
Stavropol_4_1	Potato	VINODEL	2019.05.25	90	1.778	1.336	42,148
Stavropol_4_3	Potato	VINODEL	2019.05.25	90	1.778	1.336	35,326
Stavropol_4_9	Potato	VINODEL	2019.05.25	90	1.778	1.336	42,148
Stavropol_2_0	Potato	VINODEL	2019.05.25	90	1.874	1.336	30,283
Stavropol_2_2	Potato	VINODEL	2019.05.25	90	1.874	1.336	33,225
Stavropol_4_0	Potato	VINODEL	2019.05.25	90	1.778	1.336	29,869

**Table 2:** Image stratification into four datasets.

Dataset Name	List of Images	Number of Plants	Number of Images
HQ1	Beet_marat_0, Stavropol_4_1, UBONN_Sb1_2015, Krasnodar, Stavropol_1_7, Stavropol_2_0	281,325	1102
HQ2	Stavropol_4_3, UBONN_Sb2_2015, Krasnodar_1, Stavropol_2_2	170,629	721
HQ3	Beet_marat_1, Stavropol_2_7, Stavropol_4_9, UBONN_Sb3_2015, Stavropol_4_0	186,173	459
LQ	BW_C_2021, DSC_SbCSf_2023, FYXDDS_C_2023, HZH_C, NWE_C_2022, NWE_Sb1_2022, NWE_Sb2_2022, NWE_Sf_2022, SEV_Sb_2022, UFMS_C_2023, URLTBK_P_2024, USM_T_2023, VW_C_2022, VW_Sf_2022	334,170	7453

**Table 3:** A description of the experiments conducted during the analysis.

Experiment	Training Sample	Validation Sample	Test Sample	Encoder Architecture
RN18-HQ	HQ1	HQ2	HQ3	ResNet-18
RN18-LQ	LQ	HQ2	HQ3	ResNet-18
RN18-HQ-LQ	HQ1+LQ	HQ2	HQ3	ResNet-18
RN34-HQ-LQ	HQ1+LQ	HQ2	HQ3	ResNet-34
RN50-HQ-LQ	HQ1+LQ	HQ2	HQ3	ResNet-50

**Table 4:** Accuracy measures for different experiments and images from the HR3 sample. The best parameter value in a column is shown in bold, and the worst value is underlined.

Experiment	Epoch Number for the Best Model	*MAE*	*MAPE*, %	*r*	*r*_s_	*IoU*
RN18-HQ	79	96	6.22	0.9816	0.9363	**0.3753**
RN18-LQ	50	127	7.90	0.9850	0.9597	0.3166
RN18-HQ-LQ	79	105	5.57	0.9883	**0.9600**	0.3693
RN34-HQ-LQ	76	95	5.84	0.9878	0.9489	0.3600
RN50-HQ-LQ	83	**78**	**5.20**	0.9885	0.9571	0.3688

**Table 5:** Accuracy metrics of the best model for the RN50-HQ-LQ experiment with orthomosaics from test samples. The best parameter value in a column is highlighted in bold, and the worst is underlined.

Orthomosaic Image	*MAE*	*MAPE*, %	*R*	*r*_s_
Beet_marat_1	66	**1.27**	0.9734	0.9464
Stavropol_2_7	18	2.30	**0.9995**	0.9870
Stavropol_4_9	223	12.75	0.9880	0.8790
UBONN_Sb3_2015	**4**	3.19	0.9987	**0.9950**
Stavropol_4_0	79	6.51	0.9828	0.9781

## Data Availability

The original contributions presented in this study are included in the article/Supplementary Materials. Further inquiries can be directed to the corresponding author.

## Supplementary Materials

The following supporting information can be downloaded at: https://www.mdpi.com/article/10.3390/jimaging11010028/s1, “Supplementary Material.pdf” contains the following Supplementary Materials: Table S1. The field location of the crop image dataset from Russia (2019–2023); Table S2. Public datasets from Roboflow used for the analysis (accessed on 25 November 2023); Table S3. The row spacing (for different crops) used in the work to mark up images from the additional datasets (not ours); Table S4. Description of the ResNet neural network architectures for models RN18, RN34, and RN50; Table S5. Description of the texture characteristics; Table S6. Estimates of the four texture characteristics for various datasets and markups; Figure S1. Examples of field image markups for tiles with a large deviation of the GLCM mean parameter between the manual approach and the RN50-HQ-LQ network model with the Beet_marat_1 dataset.
