# Supplementary material for: Plant Detection in RGB Images from Unmanned Aerial Vehicles Using Segmentation by Deep Learning and an Impact of Model Accuracy on Downstream Analysis
Source: J Imaging. 2025 Jan 20;11(1):28. doi: 10.3390/jimaging11010028 (PMC11766541; doi:10.3390/jimaging11010028)
Supplement: Supplementary file 1 [file jimaging-11-00028-s001.zip › jimaging-3369005-supplementary.pdf]

Supplementary material for Kozhekin et al. (2025) *Journal of Imaging*

**Table S1.** Field location for the crop image dataset from Russia (2019-2023).

| Short name | Settlement | Link to Google maps | Region |
|---|---|---|---|
| MARAT | Marat village | https://maps.app.goo.gl/Pb2Pcg4DY7i456Y2A | Penza region, Russia |
| IVANOVSK | Ivanovskaya village | https://maps.app.goo.gl/NbR8YtNZpSKy98ot8 | Krasnodar region, Russia |
| NOVOMINSK | Novominskaya village | https://maps.app.goo.gl/M2uiez62UxE1ycyG7 | Krasnodar region, Russia |
| VINODEL | Vinodelnenskiy village | https://maps.app.goo.gl/tY6mbaWW8gJbSNmW8 | Stavropol region, Russia |

**Table S2**. Public datasets from Roboflow used for analysis (accessed on 25 November 2023).

| Dataset # | Dataset name in this work | Crop (s) | Number of images | Number of plants | Year | URL |
|---|---|---|---|---|---|---|
| 1 | BW_C_2021 | Corn | 246 | 18603 | 2021 | https://universe.roboflow.com/biwen-wang/final_dataset-xzkfg |
| 2 | DSC_SbCSf_2023 | Sugar beet/Corn/ Sunflower | 189 | 5647/5257/5218 | 2023 | https://universe.roboflow.com/detection-segmentation-and-classification/plant-detection-and-counting |
| 3 | FYXDDS_C_2023 | Corn | 90 | 2400 | 2023 | https://universe.roboflow.com/fyxdds-icloud-com/maize-seedling-detection |
| 4 | HZH_C | Corn | 145 | 12236 | 2022 | https://universe.roboflow.com/huazhong/d-gndmw |
| 5 | NWE_C_2022 | Corn | 1333 | 34437 | 2022 | https://universe.roboflow.com/new-workspace-evkln/21_11_30_levee_mais |
| 6 | NWE_Sb1_2022 | Sugar beet | 411 | | 2022 | https://universe.roboflow.com/new-workspace-evkln/22_02_01_center_beet-charon |
| 7 | NWE_Sb2_2022 | Sugar beet | 624 | | 2022 | https://universe.roboflow.com/new-workspace-evkln/22_02_09_faster_beet_last |
| 8 | NWE_Sf_2022 | Sunflower | 1459 | 20167 | 2022 | https://universe.roboflow.com/new-workspace-evkln/22_03_16_tournesol |
| 9 | SEV_B_2022 | Beet | 710 | 31391 | 2022 | https://universe.roboflow.com/sergen-erbay-vl4xv/cropped_pancar |
| 10 | UFMS_C_2023 | Corn | 451 | 54399 | 2023 | https://universe.roboflow.com/ufms-agndm/detection-g0uut |
| 11 | URLTBK_P_2024 | Potato | 194 | 17309 | 2024 | https://universe.roboflow.com/universidad-rltbk/detector_malezas |
| 12 | USM_T_2023 | Tobacco | 1457 | 26486 | 2023 | https://universe.roboflow.com/university-of-state-maringa/tobacco2 |
| 13 | VW_C_2022 | Corn | 55 | 35364 | 2022 | https://universe.roboflow.com/vladimir-wwstb/corn-2xipv |
| 14 | VW_Sf_2022 | Sunflower | 89 | 27042 | 2022 | https://universe.roboflow.com/vladimir-wwstb/sunflower-fo2j4 |
| 15 | UBONN_Sb1_2015* | Sugar beet | 1 | 9544 | 2015 | https://www.ipb.uni-bonn.de/data/uav-sugarbeets-2015-16/ |
| 16 | UBONN_Sb2_2015* | Sugar beet | 1 | 28670 | 2015 | https://www.ipb.uni-bonn.de/data/uav-sugarbeets-2015-16/ |
| 17 | UBONN_Sb3_2015* | Sugar beet | 1 | 9447 | 2015 | https://www.ipb.uni-bonn.de/data/uav-sugarbeets-2015-16/ |

* - high-resolution sets, for which we collected orthomosaics and performed markup.

**Table S3.** Row spacing for different crops, used in the work to mark up images from external datasets.

| Crop | Row spacing, m |
|---|---|
| Sugar beet | 0.6 |
| Corn | 0.7 |
| Potato | 1.0 |
| Sunflower | 1.0 |
| Tobacco | 1.2 |

**Table S4.** Description of the ResNet neural network architectures for RN18, RN34 and RN50 models.

| Layer name | Output size | 18-layer | 34-layer | 50-layer |
|---|---|---|---|---|
| conv1 | 112×112 | 7×7, 64, stride | | |
| conv2_x | 56×56 | 3×3, max pool, stride 2 | | |
| | | $\begin{bmatrix} 3\times3, & 64 \\ 3\times3, & 64 \end{bmatrix}\times2$ | $\begin{bmatrix} 3\times3, & 64 \\ 3\times3, & 64 \end{bmatrix}\times3$ | $\begin{bmatrix} 1\times1, & 64 \\ 3\times3, & 64 \\ 1\times1, & 256 \end{bmatrix}\times3$ |
| Conv3_x | 28×28 | $\begin{bmatrix} 3\times3, & 128 \\ 3\times3, & 128 \end{bmatrix}\times2$ | $\begin{bmatrix} 3\times3, & 128 \\ 3\times3, & 128 \end{bmatrix}\times4$ | $\begin{bmatrix} 1\times1, & 128 \\ 3\times3, & 128 \\ 1\times1, & 512 \end{bmatrix}\times8$ |
| Conv4_x | 14×14 | $\begin{bmatrix} 3\times3, & 256 \\ 3\times3, & 256 \end{bmatrix}\times2$ | $\begin{bmatrix} 3\times3, & 256 \\ 3\times3, & 256 \end{bmatrix}\times6$ | $\begin{bmatrix} 1\times1, & 256 \\ 3\times3, & 256 \\ 1\times1, & 1024 \end{bmatrix}\times36$ |
| Conv5_x | 7×7 | $\begin{bmatrix} 3\times3, & 512 \\ 3\times3, & 512 \end{bmatrix}\times2$ | $\begin{bmatrix} 3\times3, & 512 \\ 3\times3, & 512 \end{bmatrix}\times3$ | $\begin{bmatrix} 1\times1, & 512 \\ 3\times3, & 512 \\ 1\times1, & 2048 \end{bmatrix}\times3$ |
| | 1×1 | average pool, 1000-d fc, softmax | | |
| FLOPs | | $1.8\times10^9$ | $3.6\times10^9$ | $3.8\times10^9$ |

**Table S5.** Description of the texture characteristics.

| Texture characteristic | Description | Ref. |
|---|---|---|
| Gray level co-occurrence matrix (GLCM) | $P_d(i,j) = \sum_{x=1}^{n}\sum_{y=1}^{m}\sum_{\Delta x,\Delta y}\begin{cases}1, \text{if } I(x,y)=i \text{ and } I(x+\Delta x, y+\Delta y)=j \\ \text{and } \delta(\Delta x,\Delta y)=d; \\ \text{otherwise}, 0;\end{cases}$<br>where<br>$x$, $y$ are the coordinates of pixels in the image; $0 < x < n$, $0 < y < m$; $i, j = 0, \ldots, k-1$; $k$ is the number of levels of image luminance quantization; $\delta(\Delta x, \Delta y)$, the distance between adjacent pixels $(x, y)$ and $(x + \Delta x, y + \Delta y)$; $I(x, y)$, pixel intensity in gray scale; $\Delta x$, the shift along the $X$ axis; and $\Delta y$, the shift along the $Y$ axis.<br>Normalized matrix<br>$p(i,j) = \frac{\sum_x^n \sum_y^m P_{x,y}(i,j)}{C}$,<br>where<br>$C = 2n(m-1) + 2m(n-1) + 4(n-1)(m-1)$ | [1] |

| | | |
|---|---|---|
| GLCM mean | $$\mathbf{Mean}\ \mu = \sum_{i=1}^{N_g}\sum_{j=1}^{N_g} i p_{i,j}$$ | [2] |
| GLCM correlation | $$correlation = \sum_{i=1}^{N_g}\sum_{j=1}^{N_g} p_{i,j}\left[\frac{(i-\mu)(j-\mu)}{\sigma^2}\right]$$ | [2] |
| Gray level run length matrix (GLRM) | $$R = \sum_{i=1}^{N_g}\sum_{j=1}^{N_r} q(i,j)$$ $q(i,j) = \sum_\theta Q_\theta(i,j)$, where $Q_\theta(i,j)$ is the number of runs of length $j$ of the pixels with the gray level $i$ in the direction $\theta$ from pixel $(i,j)$ | [3] |
| GLRM run length | $$Run\ length = \sum_{j=1}^{N_r}\left(\sum_{i=1}^{N_g} q(i,j)\right)^2 / R$$ | [3] |
| GLRM run ratio | $$Run\ ratio = R/\sum_{i=1}^{N_g}\sum_{j=1}^{N_r} j q(i,j)$$ | [3] |


1. Haralick, R.M.; Shanmugam, K.; Dinstein, I.H. Textural features for image classification. IEEE Transactions on systems, man, and cybernetics. 1973. 6. p. 610-621.
2. Majumdar, S.; Jayas, D. S. Classification of bulk samples of cereal grains using machine vision. Journal of Agricultural Engineering Research. 1999; 73, 1, p. 35-47.
3. Galloway, M. M. Texture analysis using gray level run lengths. Computer graphics and image processing. 1975; 4, 2, p. 172-179.

**Table S6.** Estimates of the four texture characteristics for various datasets and markups. Number of images, mean values, variances, t-statistics and *p*-values for comparison of means for manually marked plants (Mask) and neural networks RN50-HQ-LQ, RN18-HQ are shown.

| Parameter | Dataset | N | Mask | | RN50-HQ-LQ | | | | RN18-HQ | | | |
|---|---|---|---|---|---|---|---|---|---|---|---|---|
| | | | mean | var | mean | var | t | p-value | mean | var | t | p-value |
| GLCM mean | Beet_marat_1 | 81 | 14.3572 | 0.5748 | 14.1469 | 0.4723 | 3.42 | 0.0663 | 15.0020 | 1.671304 | 3.8721 | **0.00015691** |
| | Stavropol_2_7 | 121 | 4.8079 | 0.2981 | 5.9963 | 0.4819 | 14.80 | **1.16E-35** | 7.6129 | 0.947146 | 27.651 | **1.44E-76** |
| | Stavropol_4_0 | 121 | 4.7096 | 0.6764 | 6.2981 | 0.8539 | 14.13 | **2.22E-33** | 6.2162 | 0.616976 | 14.572 | **6.94E-35** |
| | Stavropol_4_9 | 121 | 6.2413 | 0.2297 | 6.0644 | 0.1626 | 3.11 | **0.0021** | 6.5512 | 0.297863 | 4.6926 | **4.53E-06** |
| GLCM correlation | Beet_marat_1 | 81 | -0.0332 | 6.71E-06 | -0.0401 | 6.48E-06 | 17.13 | **4.96E-38** | -0.0382 | 1.36E-05 | 10.037 | **1.08E-18** |
| | Stavropol_2_7 | 121 | -0.0083 | 4.98E-06 | -0.0064 | 7.12E-06 | 6.03 | **5.99E-09** | -0.0092 | 1.27E-05 | 2.4444 | **0.015232** |
| | Stavropol_4_0 | 121 | -0.0121 | 3.93E-06 | -0.0123 | 8.89E-06 | 0.67 | 0.5020 | -0.0106 | 1.46E-05 | 3.856 | **0.00014806** |
| | Stavropol_4_9 | 121 | -0.0164 | 1.26E-06 | -0.0155 | 2.77E-06 | 5.18 | **4.79E-07** | -0.0091 | 7.47E-06 | 27.134 | **4.47E-75** |
| GLRM longRun | Beet_marat_1 | 81 | 29.4053 | 0.0292 | 14.8702 | 0.0258 | 557.74 | **4.81E-265** | 14.7555 | 0.116492 | 345.37 | **9.07E-232** |
| | Stavropol_2_7 | 121 | 35.2397 | 1.4091 | 17.2543 | 0.2635 | 152.98 | **3.15E-241** | 16.1708 | 0.278859 | 161.45 | **8.54E-247** |
| | Stavropol_4_0 | 121 | 35.6687 | 3.0206 | 17.3180 | 0.4489 | 108.37 | **8.10E-206** | 17.4958 | 0.291555 | 109.84 | **3.40E-207** |
| | Stavropol_4_9 | 121 | 33.1900 | 0.2874 | 17.7510 | 0.1067 | 270.54 | **2.70E-300** | 17.3954 | 0.154089 | 261.49 | **9.21E-297** |
| GLRM runRatio | Beet_marat_1 | 81 | 0.2594 | 8.95E-07 | 0.4016 | 4.70E-06 | 540.80 | **6.66E-263** | 0.4030 | 2.00E-05 | 295.75 | **2.41E-123** |
| | Stavropol_2_7 | 121 | 0.2296 | 2.77E-05 | 0.3708 | 3.67E-05 | 193.64 | **1.33E-265** | 0.3831 | 4.38E-05 | 199.72 | **8.42E-269** |
| | Stavropol_4_0 | 121 | 0.2279 | 5.92E-05 | 0.3702 | 6.14E-05 | 142.51 | **6.41E-234** | 0.3680 | 4.01E-05 | 154.67 | **2.31E-242** |
| | Stavropol_4_9 | 121 | 0.2394 | 6.79E-06 | 0.3655 | 1.35E-05 | 308.43 | **6.4556E-314** | 0.3694 | 1.99E-05 | 276.81 | **1.13E-302** |

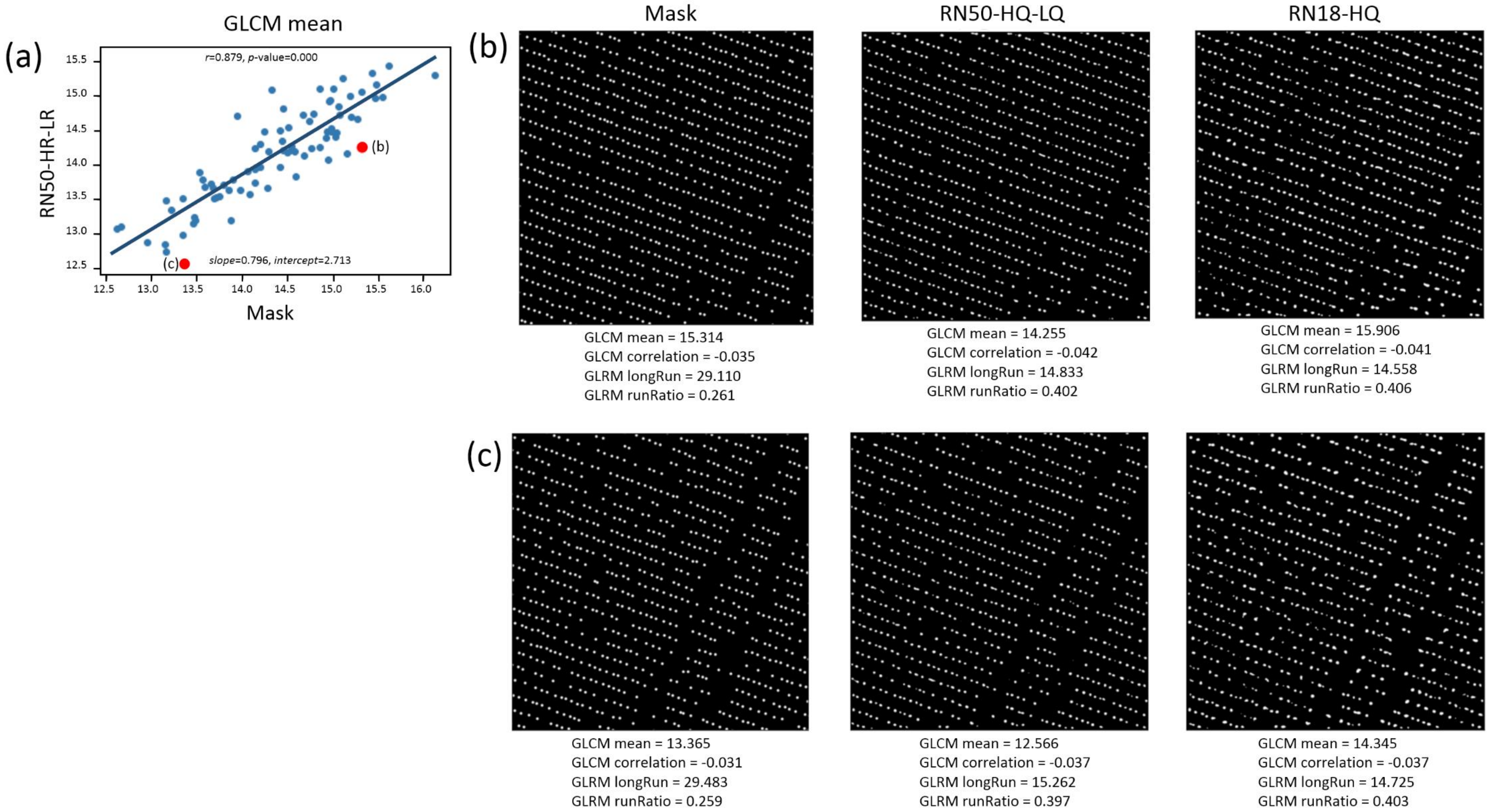


**Figure S1.** Examples of the field image markup for tiles with large deviation between GLCM mean parameter for manual and RN50-HQ-LQ network model, Beet_marat_1 dataset. (a) GLCM mean scatterplot for mask (X axis) and RN50-HQ-LQ estimates, two tiles with large deviation from y=x line are shown in red dots and panel designations. (b,c) field image markup for selected tiles from left to right: manual mask, predicted RN50-HQ-LQ and RN18-HQ masks, texture characteristics provided below images.
